# Network Properties of Electrically Coupled Bursting Pituitary Cells

**DOI:** 10.3389/fendo.2022.936160

**Published:** 2022-07-06

**Authors:** Mehran Fazli, Richard Bertram

**Affiliations:** ^1^ Department of Mathematics, Florida State University, Tallahassee, FL, United States; ^2^ Programs in Neuroscience and Molecular Biophysics, Florida State University, Tallahassee, FL, United States

**Keywords:** networks, electrical activity, pituitary, bursting, synchronization

## Abstract

The endocrine cells of the anterior pituitary gland are electrically active when stimulated or, in some cases, when not inhibited. The activity pattern thought to be most effective in releasing hormones is bursting, which consists of depolarization with small spikes that are much longer than single spikes. Although a majority of the research on cellular activity patterns has been performed on dispersed cells, the environment *in situ* is characterized by networks of coupled cells of the same type, at least in the case of somatotrophs and lactotrophs. This produces some degree of synchronization of their activity, which can be greatly increased by hormones and changes in the physiological state. In this computational study, we examine how electrical coupling among model cells influences synchronization of bursting oscillations among the population. We focus primarily on weak electrical coupling, since strong coupling leads to complete synchronization that is not characteristic of pituitary cell networks. We first look at small networks to point out several unexpected behaviors of the coupled system, and then consider a larger random scale-free network to determine what features of the structural network formed through gap junctional coupling among cells produce a high degree of functional coupling, i.e., clusters of synchronized cells. We employ several network centrality measures, and find that cells that are closely related in terms of their closeness centrality are most likely to be synchronized. We also find that structural hubs (cells with extensive coupling to other cells) are typically not functional hubs (cells synchronized with many other cells). Overall, in the case of weak electrical coupling, it is hard to predict the functional network that arises from a structural network, or to use a functional network as a means for determining the structural network that gives rise to it.

## 1 Introduction

The anterior pituitary contains five types of secretory cells: lactotrophs, somatotrophs, corticotrophs, gonadotrophs, and thyrotrophs. Each cell type is electrically active, and hormone secretion is greatly facilitated when the cell is in an electrical bursting state. This is because the relatively long duration of a burst brings enough Ca^2+^ into the cell to significantly elevate the intracellular Ca^2+^ concentration and thereby evoke hormone secretion (1–4). Thus, when the endocrine pituitary cells are bursting they are actively secreting hormone, but while in a silent or tonic spiking state they are not (or the hormone secretion rate is at a low level). There are two general classes of bursting produced by these cells, generated through very different mechanisms. In gonadotrophs, long “plateau bursts” are produced when the cells are activated through the release of Ca^2+^ from intracellular stores and its action on Ca^2+^-activated K^+^ channels (5). In the other cell types, shorter “pseudo-plateau bursts” are produced by ionic current through plasma membrane ion channels and feedback from intracellular Ca^2+^ onto SK– and BK–type K^+^ channels (1, 6–8).

Much of what is known about pituitary cell biophysics is based on studies of dispersed cells (2). However, *in situ*, the various cell types form networks in which cells are electrically coupled through gap junctions (9–12). Studies using Ca^2+^ imaging with fluorescent dyes have demonstrated that these “structural networks” of electrically-coupled cells produce “functional networks” of cells whose Ca^2+^ oscillations, which are produced through pseudo-plateau bursting in activated cells, are synchronized (9). Thus, the electrical coupling among cells of the same type impacts their electrical activity *in situ* in a way that is most readily monitored through Ca^2+^ imaging in pituitary slices. What can such a functional network, where nodes are cells and edges represent coincident Ca^2+^ oscillations, tell us about the underlying structural network, where the edges represent electrical coupling? A major aim of this study is to answer this question by determining how properties of the structural network impact the resulting functional network.

We begin this computational study with the simplest case of two electrically coupled identical pseudo-plateau bursters. How does the coupling affect their bursting pattern? Does electrical coupling induce synchronization of the bursts? We then move to a larger, but still small, regular network of identical pseudo-plateau bursting cells to gain additional insight into synchronization properties of electrically coupled pseudo-plateau bursters. Finally, we move to still larger networks that have a random coupling pattern, but that have a power-law degree distribution. This choice of degree distribution is based on a prior experimental study that found that functional networks of lactotrophs have such a distribution (13, 14). Networks with a power-law degree distribution, also called “scale-free networks”, consist of many nodes with low degree (in our case, the degree is the number of other cells that a cell is electrically coupled to) and include a few nodes with much higher degree called “hubs”. We ask whether structural hubs are also functional hubs, that is, whether bursting oscillations in the structural hub cells are synchronized with those of many other cells in the network. We also ask the converse question of whether functional hubs are structural hubs. That is, if a cell is synchronized with many others, does that mean that it is electrically coupled to many others?

Overall, we find that the behaviors exhibited by networks of coupled pseudo-plateau bursters can be counter-intuitive. In particular, it is hard to draw information about the structural network from the functional network, and attempts at this can be very misleading. However, all is not lost, as there are general principles that can help one to interpret what functional network properties say about the underlying structural network. These insights should be useful in the interpretation of data in future studies of oscillations in pituitary slices, and in future theoretical investigations of how single-cell electrical activity is coordinated in a physiological setting in which the cells are enmeshed in electrically coupled networks.

## 2 Materials and Methods

The mathematical model describes electrical activity in pituitary lactotrophs, and has low dimensionality (15). The variables are the membrane potential *V*, the free cytosolic Ca^2+^ concentration *c*, and activation variables *n* and *b* for delayed rectifier potassium (K) and big conductance potassium (BK) channels, respectively. The variable dynamics are governed by the differential equations


(1) $$C_{m}\frac{dV}{dt}=-\left(I_{\text{Kdr}}+I_{\text{Ca}}+I_{\text{BK}}+I_{\text{SK}}+I_{\text{L}}+I_{\text{c}}\right),$$

(1)

$\tau_{n}\frac{dn}{dt}=n_{\infty }\left(V\right)-n,$ (2)

$\frac{dc}{dt}=-f_{c}\left(\alpha I_{\text{Ca}}+k_{c}c\right),$ (3)

$\tau_{b}\frac{db}{dt}=b_{\infty }\left(V\right)-b,$ (4)

where *C_m_* is the membrane capacitance. The ionic currents are: *I*_Kdr_(delayed rectifier K ^+^ current), *I*_Ca_ (L-type Ca^2+^ current), *I*_BK_(big conductance K+ current), *I*_SK_(small conductance K^+^ current) and *I*_L_(leak current), and all are produced by the flux of ions between the cytosol and extracellular spaces through open ion channels. In contrast, the electrical coupling current, *I_c_*, is produced by the flux of ions between neighboring cells through gap junctions. The parameter *f*_*c*_ is the fraction of cytosolic Ca^2+^ that is free, while *k*_*c*_ denotes the rate of pumping through Ca^2+^ pumps in the plasma membrane, and *α* is the conversion from Ca^2+^ current to concentration. The time constants for activation of the K^+^ channels are denoted by *τ*_*n*_ and *τ*_*b*_. The equations for ionic currents are:

*I*_Kdr_=*g*_Kdr_ *n*(*V*−*V*_K_), (5)

*I*_Ca_=*g*_Ca_ *m*_*∞*_(*V*)(*V*−*V*_Ca_), (6)

*I*_BK_=*g*_BK_ *b*(*V*−*V*_K_), (7)

*I*_SK_=*g*_SK_ *s*_*∞*_(*c*)(*V*−*V*_K_), (8)

*I*_L_=*g*_L_(*V*−*V*_L_), (9)

$I_{\text{c}}=\sum_{j\in N_{i}}g_{c}\left(V_{i}-V_{j}\right),$

(10)where *g_x_* is the maximal conductance for *x*∈{Kdr,Ca,BK,SK,L} and *g_c_* is the electrical coupling conductance mediated by gap junctions. The appropriate units for conductance are nS, but for convenience we report the coupling conductance in units of pS. Also, *V_x_* (for *x*∈{K,Ca,L}) is the Nernst potential of each corresponding current. The equilibrium functions for gating variables, *x*_*∞*_(*V*) (for *x*∈{*n*,*b*,*m*}), are defined by:

$x_{\infty }\left(V\right)=\frac{1}{1+\text{exp}\;\left(\frac{\nu_{x}-V}{l_{x}}\right)},$ (11)

where *ν*_*x*_ is the half-activation membrane voltage and *l*_*x*_ is the slope factor for the function. The fastest gating variable, *m*, is assumed to be at quasi-equilibrium, so *m*=*m*_*∞*_(*V*). Similarly for the SK channel activation variable *s*, but this is gated by the cytosolic Ca^2+^ according to:

$s_{\infty }=\frac{c^{2}}{c^{2}+k_{\text{SK}}^{2}}.$ (12)

Finally, in equation (10), *N*_*i*_ denotes the set of electrically-coupled neighbors of cell *i*.

To check for synchronization of oscillations between two cells, we use the metric employed by (13) and compute a similarity value *S*_*ij*_ between cells *i* and *j*:

$S_{ij}=\frac{T_{ij}}{\sqrt{T_{i}T_{j}}},$ (13)

where *T*_*i*_ and *T*_*j*_ are the active phase durations of each cell and *T*_*ij*_ is the length of time that cells *i* and *j* are in their active phases simultaneously. These duration values are computed during the last 10 seconds of a simulation that is sufficiently long so that transients are removed.

All parameter values are given in **Table 1**, and with these values the model produces a pseudo-plateau bursting pattern. Computer simulations were performed using the Runge-Kutta method in Python with time step Δ*t*=0.5 ms. Computer code can be downloaded from www.math.fsu.edu/bertram/software/pituitary.

**Table 1:** Model parameter values for each model lactotroph.

Name	Value	Name	Value
*C_m_*	5 pF	*v_n_*	–5 mV
*g*_Kdr_	2.5 nS	*v_m_*	–20 mV
*g*_Ca_	2.1 nS	*v_b_*	–5 mV
*g*_L_	0.2 nS	*l_n_*	1 mV
*g*_SK_	2 nS	*l_m_*	12 0mV
*g*_BK_	1 nS	*l_b_*	2 mV
*g_c_*	0.002nS	*α*	0.0015 *μ* M/fC
*V*_ca_	60 mV	*f_c_*	0.005
*V*_K_	–75mV	*k_c_*	0.12 *μ* M
*V*_L_	–50 mV	*k*_SK_	0.4 *μ* M
*τ_n_*	30 ms	*τ_b_*	5 ms

## 3 Results

### 3.1 Synchronization Properties of a Pair of Coupled Pseudo-Plateau Bursters

**Figure 1:**
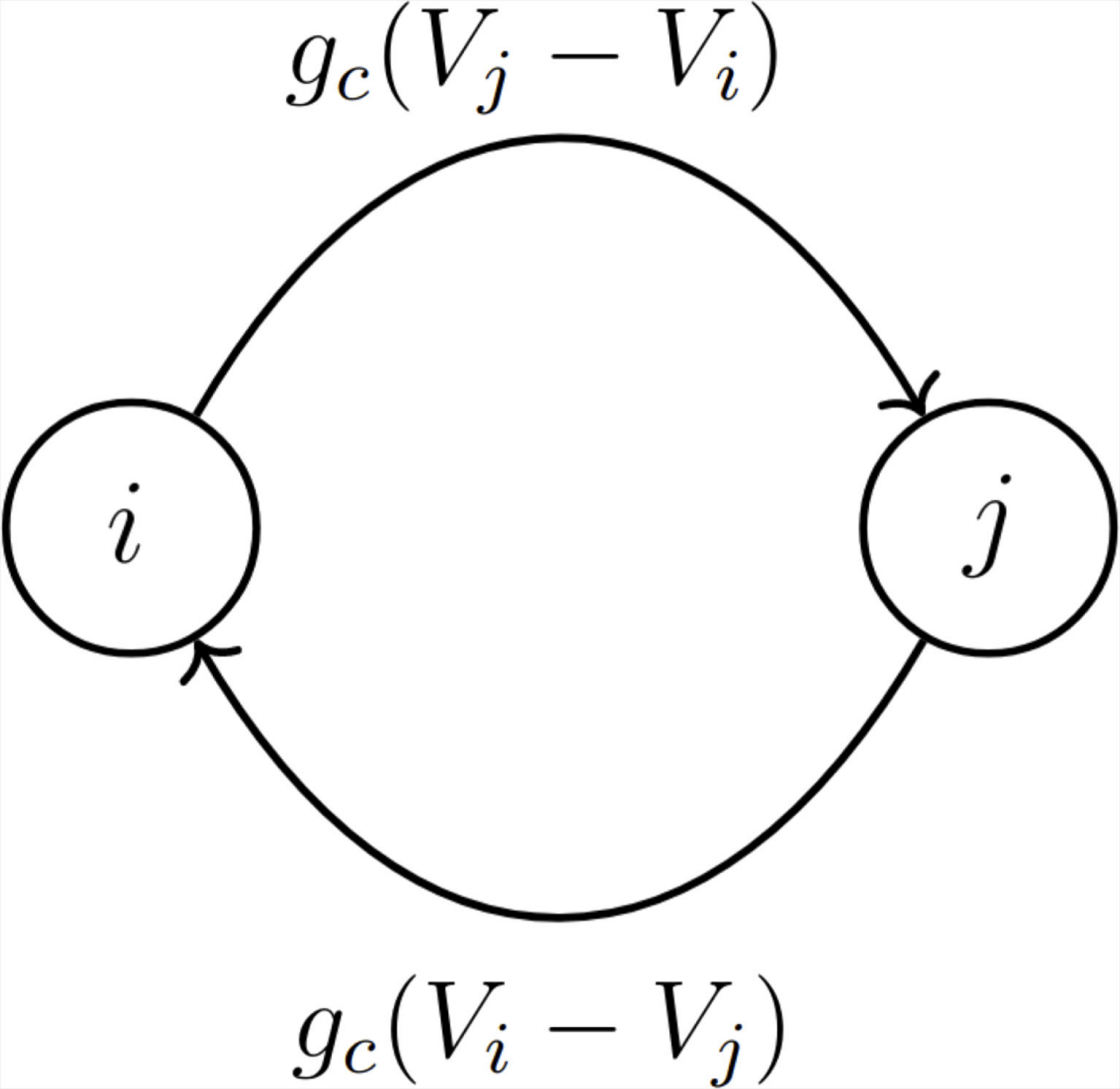
Two model pituitary lactotrophs, denoted as cells *i* and *j*, are bidirectionally coupled with coupling conductance *g*_*c*_ and a total coupling current of magnitude *g*_*c*_|(*V*_*i*_−*V*_*j*_)| .

**Figure 2:**
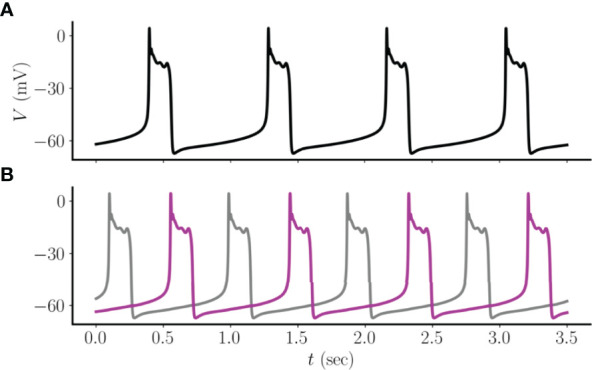
**(A)** Two electrically coupled pseudo-plateau bursters approach a synchronized asymptotic state when starting from one set of initial conditions. **(B)** For the same set of parameters, but a different set of initial conditions, the two coupled cells approach a state in which the oscillations are in antiphase. In this example, *g*_*c*_=2 pS.

The data showing coupled networks of endocrine cells (lactotrophs and somatotrophs) within the pituitary (9, 16) motivates our study of the synchronization properties of electrically coupled model pituitary cells exhibiting pseudo-plateau bursting (15). We begin with the case of two bidirectionally coupled cells, where the coupling is through gap junction proteins. These proteins provide symmetric electrical coupling between the cells (**Figure 1**), with a coupling conductance or strength given by the parameter *g_c_* (see Materials and Methods for full model description). Since the coupling current, *I*_*c*_=*g*_*c*_(*V*_*i*_−*V*_*j*_), is largest when the cells are at very different voltages and has polarity such that the current brings cells together, it is not surprising that a tendency is for the cells to synchronize (**Figure 2A**), minimizing the electrical potential difference between them. However, there is a second stable state in which the cells exhibit antiphasic pseudo-plateau oscillations. In this case, the active phase of the burst in one cell, where the membrane potential *V* and Ca^2+^ concentration *c* are elevated, is 180^o^ out-of-phase with that of the other (**Figure 2B**). A similar behavior was first described in a study of two electrically coupled cells exhibiting tonic spiking (17). That study also showed that if the cells exhibit “plateau” or “square-wave” bursting, then with weak coupling the bursts synchronize, but the spikes within the bursts have an antiphase pattern. The dynamic mechanism for plateau bursting is, however, very different from that of pseudo-plateau bursting (18), and we demonstrate here that the coupling of pseudo-plateau bursts can in fact result in stable antiphase oscillations.

**Figure 3:**
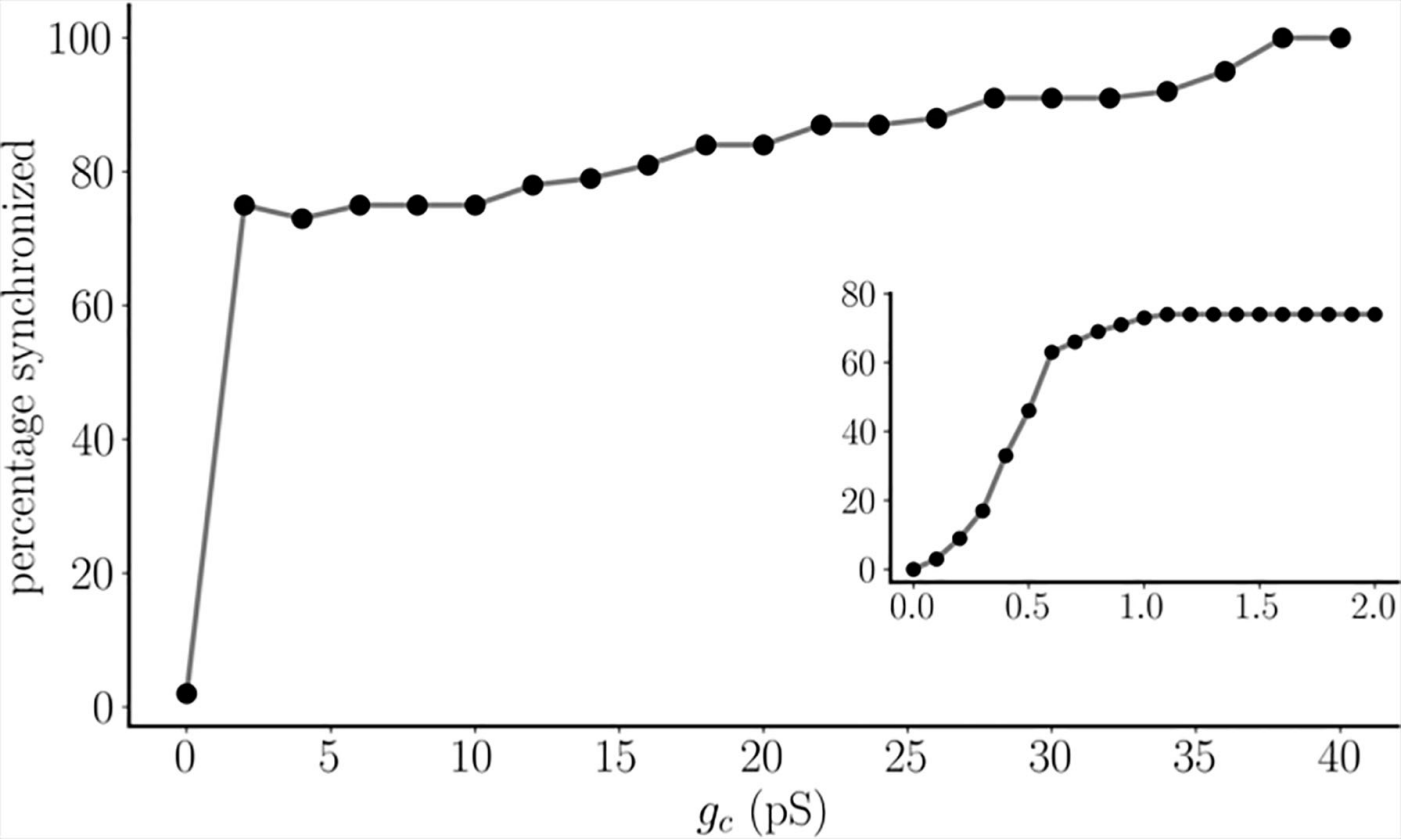
The percentage of synchronized pseudo-plateau bursting oscillations for a pair of identical model pituitary cells. For each coupling strength, *g*_*c*_ , simulations are run for 100 randomly-determined initial conditions. This same set is used for each value of *g*_*c*_ . For the main graph, the coupling strength step size is 2 pS, but a smaller step size of 0.1 pS is used for the inset. Note that units of pS are used in the figure.

There is a range of coupling strengths where both stable states are possible. That is, there is an interval of *g*_*c*_ values where the coupled system is bistable. Over this interval, the final state of the system, synchronous or antiphasic oscillations, depends on the choice of initial conditions. To explore this, we performed 100 simulations for each *g_c_* value over a range from *g_c_* = 0 to *g_c_* = 40 pS. In each of the 100 trials a different set of initial conditions was used from a random sampling of all model variables. This same set of initial conditions was used in simulations with each *g_c_* value. After performing simulations of sufficient length to remove transients, the asymptotic state of the system was then determined. This is quantified in **Figure 3**, where for each value of *g_c_* we report the fraction of trials in which a synchronous asymptotic state was achieved (we show the coupling conductance in units of pS here for visual clarity; the units used in model simulations are nS). When there is no coupling, there was no synchronization, as expected. At the lowest coupling strengths explored, there is a rapid increase in the percentage of cells synchronized (inset) that flattens out by *g_c_ = 1* pS, with over 70% of the trials synchronized (as in **Figure 2A**), while the remainder resulted in antiphasic oscillations (as in **Figure 2B**). From here, the percentage of synchronous oscillations increased slowly but steadily with an increase in coupling strength, reaching 100% by *g_c_* = 40 pS. Because the initial conditions were chosen randomly, these large fractions in the synchronized state suggest that the basin of attraction of the synchronous state is larger than that of the antiphasic state over the set of coupling strengths that we examined, and that this dominance grows with the coupling strength until the antiphasic state becomes very rare. However, the antiphase equilibrium state remains, since cells that are in antiphase with a low coupling strength remain in that state when the coupling strength is increased to the largest value examined, *g_c_* = 40 pS. It appears, then, that both asymptotic solutions are present and are stable over the entire range of coupling values examined, though the synchronous state dominates the antiphasic state with larger coupling.

### 3.2 Synchronization Properties of Small Center/Satellite Networks of Coupled Pseudo-Plateau Bursters

**Figure 4:**
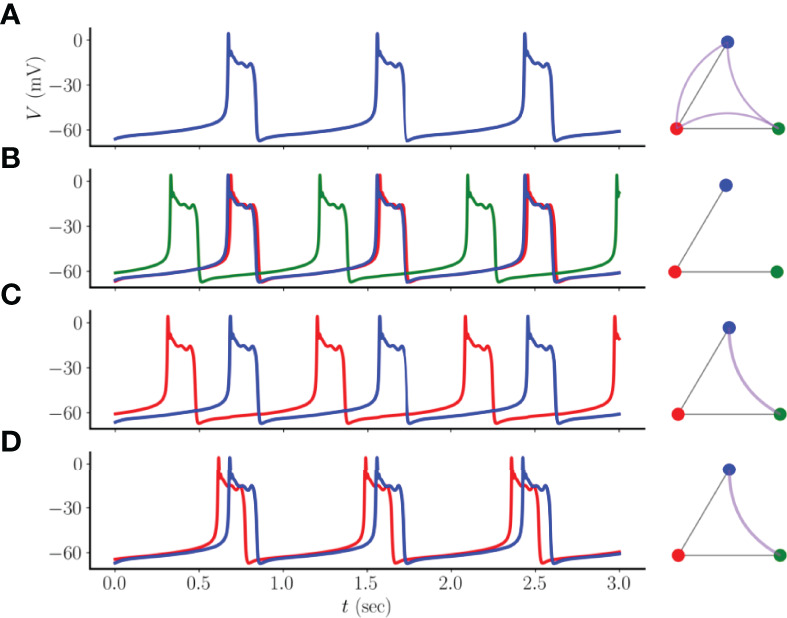
Four asymptotic states that can arise from a three-node network consisting of a center cell (red node) coupled to two satellite cells (blue and green). **(A)** A homogeneous state in which bursting is perfectly synchronized across all cells. In the network graph on the right, straight edges indicate electrical or structural coupling, while curved edges indicate synchrony or functional coupling. The functional network graph is complete. **(B)** The center cell is nearly synchronized with a satellite, while the other satellite is far out of phase. There are no edges in the functional network. **(C)** The two satellite cells are synchronized, while the center cell is far out of phase. The single edge in the functional network is not an edge in the structural network. **(D)** Similar to the previous panel, but bursting in the center cell largely overlaps that of the satellites. The coupling conductance is *g*_*c*_=2 pS.

We next examine the case in which a cell (which we refer to as the “center cell”) is electrically coupled to two others (which we refer to as “satellite cells”), so that its degree (the number of edges in the “structural network”) is twice that of the neighboring cells. How does this asymmetry affect the asymptotic states of the system? To answer this question, we again performed 100 simulations with randomly chosen initial conditions, all with the same coupling conductance of *g_c_* = 2 pS. We found four types of asymptotic states (**Figure 4**). In the first, the three cells are in a perfectly synchronized asymptotic state. This is shown in **Figure 4A**, along with an illustration of the simple network, where each colored node represents a pseudo-plateau bursting cell (red indicates the center cell), straight edges represent electrical coupling, and curved edges indicate cell synchrony. This network diagram therefore represents both the structural network, where the edges reflect electrical coupling, and the “functional network”, where the edges reflect perfect synchrony. In this case, the functional network is complete, containing all possible functional edges. In a different asymptotic state the opposite occurs, where there are no edges in the functional network (panel B). For this asymptotic solution, one of the satellite cells is nearly-synchronous with the center cell, while the other is far out of phase with it. Although there is a high degree of overlap in the burst of the nearly-synchronous center cell and a satellite cell, it is not total overlap, so no functional edge is drawn between the two cells. (Later, we soften the synchrony condition to include almost-synchronous cases like this).

The last two asymptotic states show cases in which the satellite cells are synchronized with each other, but not with the center cell. In one case, the center cell is far out of phase from the satellites (panel C), while in the other case there is substantial overlap of the center and satellite bursts (panel D). This may seem counter-intuitive, since the satellites are not electrically coupled, yet they are synchronized. In contrast, the one cell that is coupled to both is the one that is not synchronized. Thus, even with this simple three-node example, it is evident that the functional network that reflects cell synchrony can have little relation to the structural network that underlies the synchronization.

**Figure 5:**
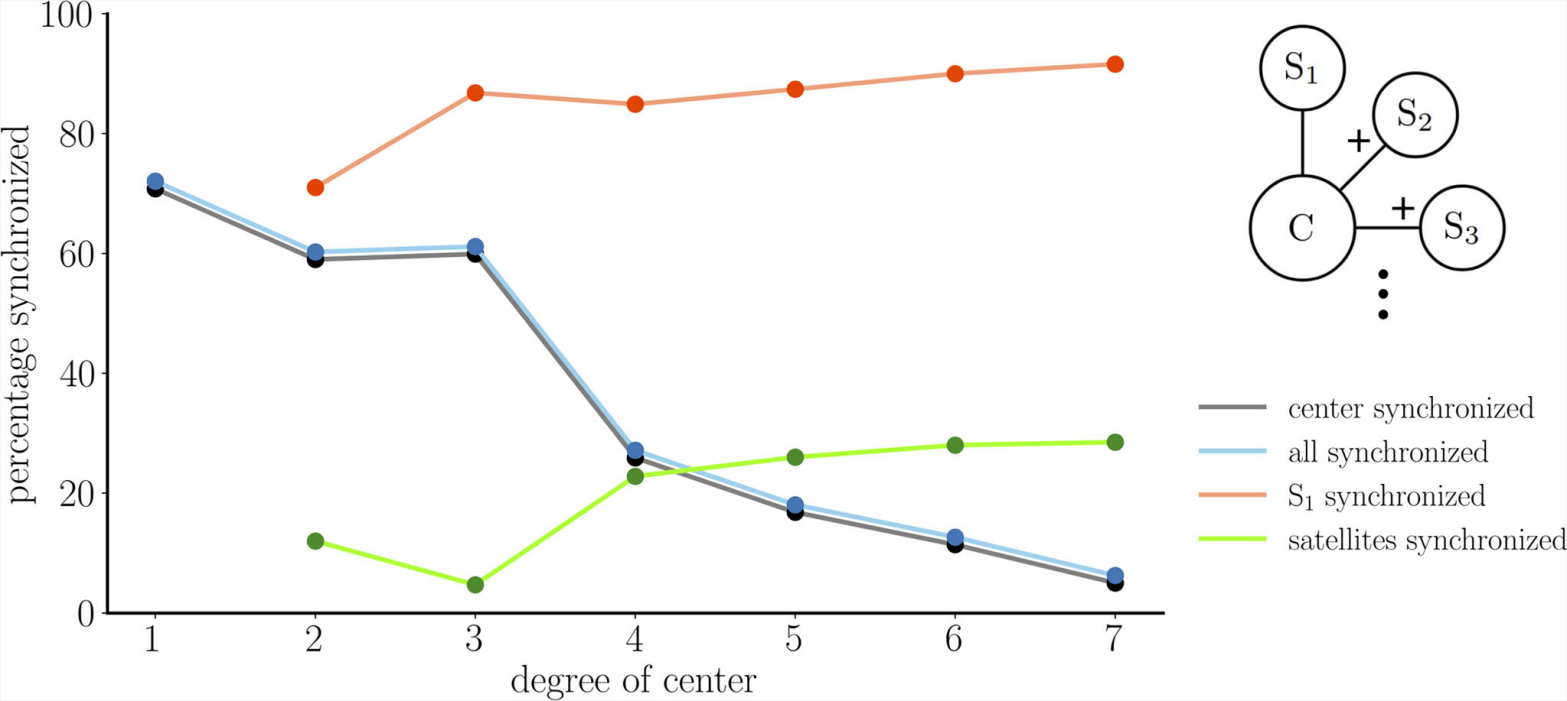
Results from center/satellite networks as the number of satellites, or the degree of the center node, is increased. The percentages of synchronized cells was based on 1000 trials in which initial conditions were chosen randomly. The cyan curve represents asymptotic states in which the center cell is synchronized with all satellites, which is identical to the number of trials in which the center is synchronized with at least one satellite (gray curve, shifted down for visibility). The green curve represents asymptotic states in which the satellites are all synchronized, but not with the center cell. The red curve represents cases in which satellite cell S _1_ is synchronized with other cells. The latter two curves begin at degree=2 since this is when the structural distinction between “satellite” and “center” cells first occurs.

One interpretation of **Figure 4** is that with coupling conductance of *g_c_* = 2 pS there is one state in which the center cell is perfectly synchronized with the satellites and three states where it is not. In **Figure 5** we examined how often the center cell synchronizes with the satellites as the number of satellites is increased from 1 to 7. For each configuration, 1000 simulations were performed, each started from random initial conditions. With a single satellite, the center and satellite are synchronized more than 70 % of the time (as shown in **Figure 3**). With two satellites, the center synchronizes with the satellites ∼60 % of the time (cyan curve). In an additional ∼10 % of the simulations, the satellites synchronize, but not the center cell (green). As the number of satellites (or degree of the center node) is increased, the percentage of simulations in which all cells synchronize declines, to ∼5 % of the simulations when degree=7, while the percentage of simulations in which the satellites synchronize, but not the center, increases to ∼25 %. Thus, if the degree of the center node is 5 or greater, it is more likely that the satellites synchronize without the center node than it is that they synchronize with the center node. This is in spite of the fact that the satellites are not directly coupled; coupling only occurs through the center node.

Synchronicity between two cells is synonymous with a link between these nodes in the functional network. With this center/satellite network structure, what fraction of the time is the center cell connected to others in the functional network? What fraction of the time is a randomly chosen satellite cell connected in the functional network? These questions are also addressed in **Figure 5**. The gray curve shows the percentage of the 1000 simulations in which the center cell is synchronized with the other cells. That is, the percentage of simulations resulting in an edge between the center node to other nodes in the functional network. This curve is identical to the cyan curve since if the center is synchronized with one satellite it is synchronized with them all. The curve declines as the number of satellites is increased. The red curve indicates the percentage of time that an arbitrary satellite cell, denoted as S_1_, has an edge in the functional network. This starts at ∼70 % when there are two satellites and increases to ∼90 % when there are 7 satellites. Thus, satellites are much more likely to be connected in the functional network than is the center cell, and this effect is amplified as the number of satellites is increased.

### 3.3 Synchronization Properties of a Small Multi-Arm Network of Coupled Pseudo-Plateau Bursters

**Figure 6:**
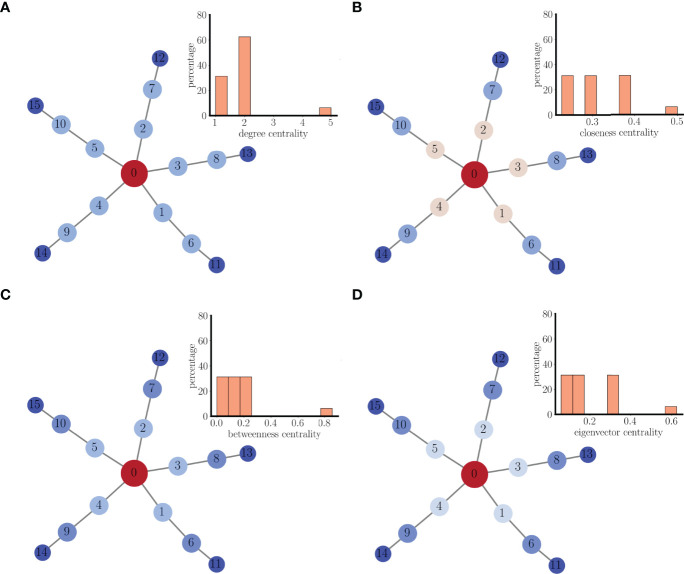
A multi-arm structual network of coupled pseudo-plateau bursters. **(A)** Nodes are colored according to degree centrality. The inset is a histogram of the centrality for all nodes in the network. **(B)** Nodes are colored according to closeness centrality. **(C)** Nodes are colored according to betweenness centrality. **(D)** Nodes are colored according to eigenvector centrality.

An extension of the center/satellite networks discussed above is one in which each satellite is one node on an arm of nodes that extends from the center node, as shown in **Figure 6**. The nodes in this network can be characterized in terms of their centrality to the structural network. There are, however, many centrality metrics that are often used (19). The simplest is degree centrality, which is the degree of the node. In **Figure 6A** the nodes are colored according to their degree centrality, and the associated histogram shows the percentages of nodes with different centrality values. The center node has the highest centrality, so is colored red. The next two nodes on each arm have lower centrality values, but are all the same. Finally, the nodes farthest out on the arms have the lowest degree centrality of 1. In panel B, each node is colored according to its closeness centrality; the reciprocal of the sum of the distances (in terms of edge number) between that node and every other node. The center node has the highest closeness centrality (it is closest to the other nodes) and nodes on each concentric ring have the same closeness centrality, declining with more distant rings. In panel C, each node is colored according to its betweenness centrality; the fraction of shortest paths that go through that node. Again, the center node has the highest betweenness centrality, and the value is uniform on each ring, but declines with more distant rings. Finally, in panel D, each node is colored according to its eigenvector centrality; a measure that counts edge number but weights each edge according to the centrality of the node that it connects to. Once again, the center has the highest centrality value, nodes on a ring have identical centrality values, and the value declines with distance from the center.

**Figure 7:**
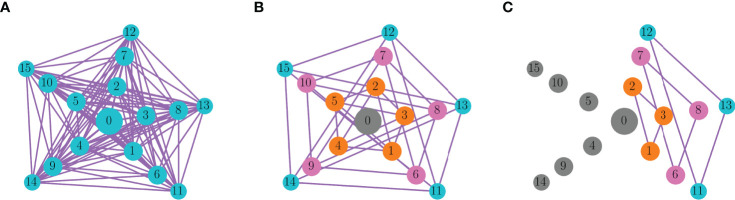
Functional network graphs for a multi-arm network of pseudo-plateau bursters. Nodes of the same color are synchronized, and the edges indicate synchronization of the connected nodes. Gray nodes are not synchronized with any other node. **(A)** Of the 100 trials that start from randomized initial conditions, 26 resulted in complete synchronization of the network. **(B)** In 41 trials, each ring of nodes (the same distance from the center cell) synchronized, but nodes in different rings did not. **(C)** In 33 trials, subgraphs of the rings were synchronized. This graph shows one such example.

The rationale for considering the centrality measures of the nodes is to attempt to form a correspondence between centrality and the synchronization properties of the network. In particular, to determine if some form of centrality provides insight into the connectivity of the functional network. To begin to investigate this, we performed 100 simulations from randomized initial conditions using the mutli-arm network (with coupling conductance *g*_*c*_=2 pS). In 26 trials, the entire network synchronized, so the functional network consists of a complete graph (**Figure 7A**). In 41 trials, there was functional connectivity among all nodes of each ring. That is, nodes the same distance from the center synchronized, but nodes at different distances did not (**Figure 7B**). This is similar to the distribution of closeness, betweenness, and eigenvector centrality, but not degree centrality (**Figure 6**). Thus, the structure of the functional network did not match the degree distribution of the nodes, indicating that degree distribution may not be a reliable predictor of functional network connectivity. Finally, in 33 trials, a subset of nodes on each ring synchronized, but not all ring elements. Panel C shows one example of this. Nodes 1, 2, and 3 of the innermost ring were synchronized, for example, but nodes 4 and 5 of that ring were not. Thus, there is synchronization of subgraphs of the rings. This demonstrates that the other centrality measures were only somewhat reliable in predicting functional connectivity; they correctly predicted that nodes on different rings should not be synchronized, but incorrectly predicted that all nodes on the same ring should be synchronized. However, these centrality measures are clearly better than degree centrality in predicting functional network structure.

**Figure 8:**
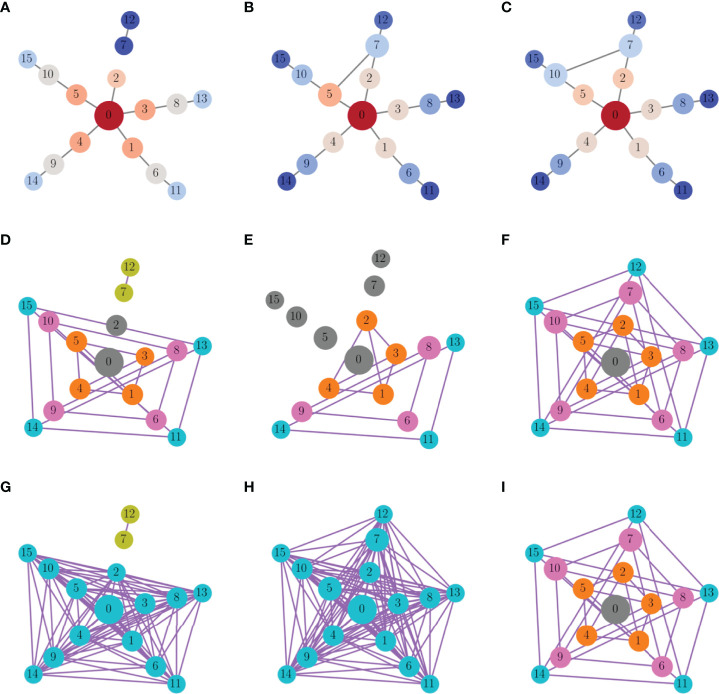
The effects of removing or adding an edge to the multi-arm network. The functional networks in the second and third row correspond to the structural networks on the top row. **(A)** The edge between nodes 2 and 7 was removed. Nodes are colored according to closeness centrality, and the edge removal changes centrality of nodes 2, 7, and 12. **(B)** An edge was added between nodes 5 and 7, changing closeness centrality of nodes 5, 7, and 10. **(C)** An edge was added between nodes 7 and 10, which are the same distance from the center node. This changes closeness centrality in nodes 2, 5, 7, and 10. **(D–F)** Asymptotic states of the functional network starting from initial conditions corresponding to the ring-synchronized state of **Figure 7B**. **(G–I)** Asymptotic states of the functional network starting from initial conditions that produced the ring-synchronized state of **Figure 7B**.

How does the ring-like connectivity pattern of the functional network (**Figure 7B**) change if the symmetry of the structural network is broken? We explored this question by either removing or adding an edge to the multi-arm network. As a first example, we removed an edge connecting nodes 2 and 7. This single change affected the closeness centrality of nodes 2, 7, and 12, as reflected in the color coding in **Figure 8A**. When the model cells were then started from the ring-synchronized asymptotic state of **Figure 7B** and run until a new asymptotic state was reached, that state produced the functional network graph of **Figure 8D**. Comparing this functional network with that prior to edge removal (**Figure 7B**) it appears that the rings remained synchronized, except for the nodes whose closeness centrality changed due to edge removal. Node 2 became unsynchronized with other nodes, while nodes 7 and 12 synchronized with each other. However, when the same initial conditions were used that previously resulted in ring-like synchronization (i.e., nodes on the same ring are synchronized, but not with nodes of different rings), then with the deletion of a single edge the network became fully synchronized, except for nodes 7 and 12 which synchronized with each other (**Figure 8G**). Thus, the trajectory from initial conditions to the asymptotic state of the functional network was quite sensitive to the removal of a single edge in the structural network.

Rather than removing an edge, an edge was added between nodes 5 and 7 in the next example. This changed the closeness centrality of three nodes: numbers 5, 7, and 10 (**Figure 8B**). As was done with the edge removal example, the new network was simulated first with initial conditions corresponding to the ring-synchronized asymptotic state (**Figure 7B**). The resulting functional network had synchronization within subgraphs of the rings (**Figure 8E**), as described in **Figure 7C**. The three nodes whose closeness centrality was changed by the edge addition became desynchronized, as were several other nodes. When the system was started with initial conditions that led to a ring-synchronized state in the original network, the system now evolved to a fully synchronized state with the addition of the edge (**Figure 8H**).

In the final example, an edge was added between nodes 7 and 10, changing the centrality of four nodes: 2, 5, 7, and 10. This change in the structural network, connecting two nodes on the same ring, had no impact on the functional network for either set of initial conditions (**Figures 8F, I**). This is perhaps not surprising, since the new edge connected nodes that would synchronize anyway in the ring-synchronization state, so this maneuver just reinforced the synchronization that would have occurred anyway. Overall, these three examples demonstrate the variation in impact that a single change to the structural network can have on network synchronization.

### 3.4 What Determines the Functional Hubs?

**Figure 9:**
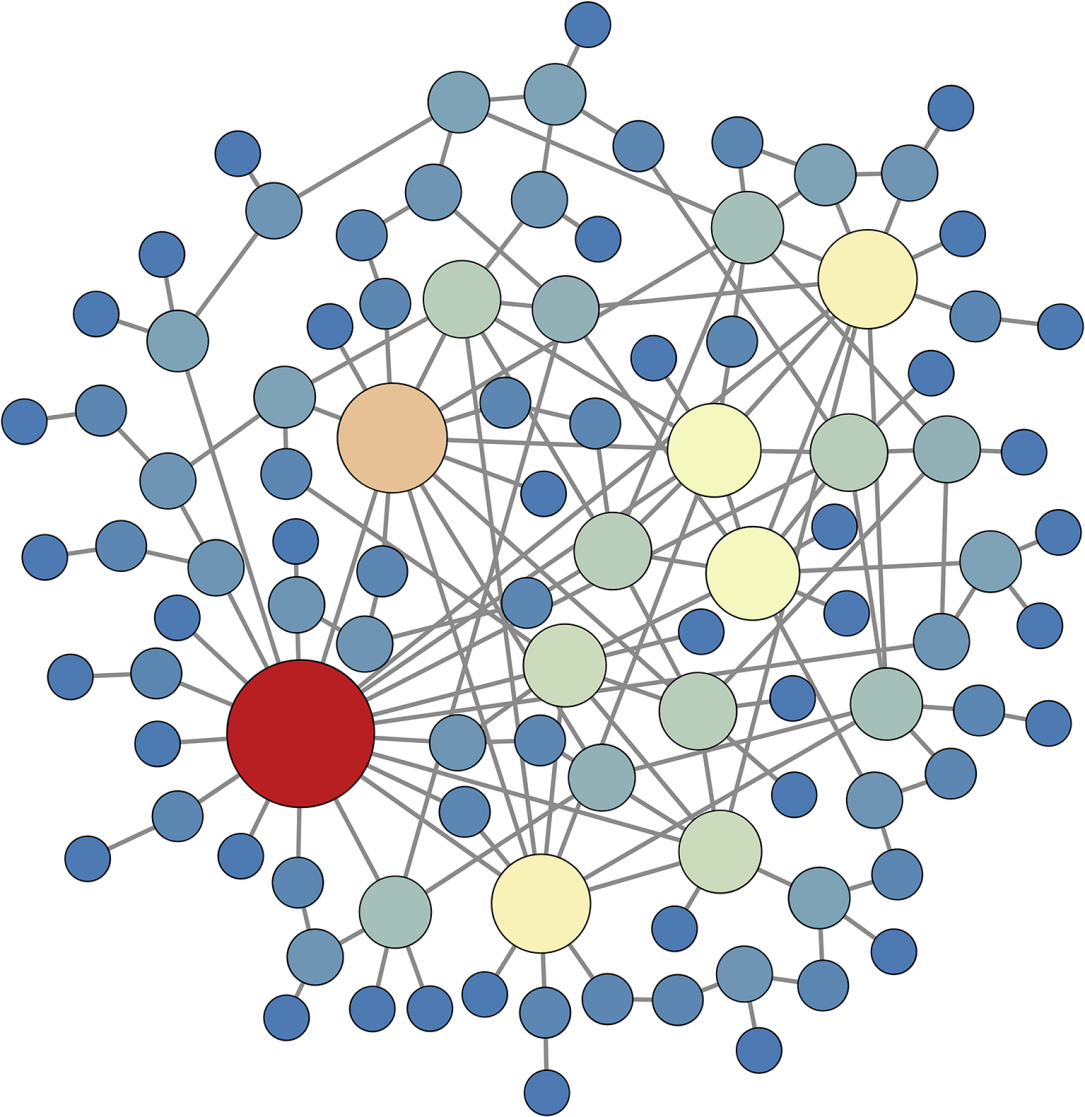
A random network that satisfies a power law degree distribution with exponent *γ*=2.8 , constructed using a configuration model algorithm. Large nodes with hotter colors have high degree. The yellow, orange, and red nodes can be considered to be structural hubs. This structural network is used in simulations for subsequent figures.

Functional hubs are nodes with the highest degree in the functional network, and are thus cells that synchronize with a large number of other cells. What properties of the structural network give rise to such highly synchronized cells? To address this question, we turn to a larger network, with 100 nodes, where the connectivity pattern is random. We construct the network of pseudo-plateau bursting cells so that the degree probability distribution satisfies a power law: *p*(*k*)∝*k* ^−*γ*^ with γ=2.8. Networks satisfying a power law degree distribution are often called “scale-free”, and consist of many nodes with a low degree, but also a smaller subpopulation of nodes with considerably higher degree, i.e., hubs. An example of such a network is shown in **Figure 9**, where the degree of each node is reflected in both the size of the node and its color (large nodes with hotter colors have the highest degree). In this example the red, orange, and yellow nodes could be considered hubs. The edge placement is determined using a configuration model algorithm (19).

**Figure 10:**
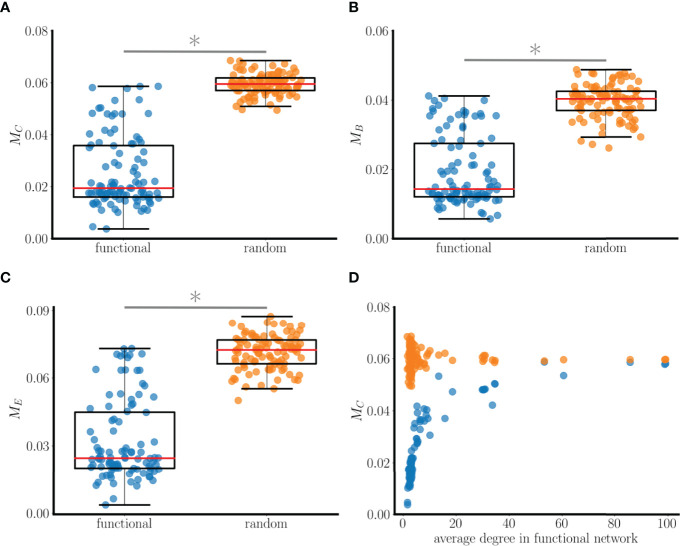
Relationship between connectedness in a 100-node functional network and various centrality measures. The functional networks are based on 100 simulations with random initial conditions using the scale-free structural network of **Figure 9**. **(A)** *M*_*C*_ (closeness centrality difference) values computed from functional networks (blue points) and from random baseline networks (orange points). Low values mean similarity of closeness values in the structural network between neighboring nodes of the functional network. The median value of *M*_*C*_ for the functional network is roughly a third that of the baseline network, and the difference in the distributions is significant (*: p<0.001 for significance, Wilcoxon test). **(B)** Comparison using a betweenness centrality metric *M*_*B*_. **(C)** Comparison using an eigenvector centrality metric *M*_*E*_. **(D)** The *M*_*C*_ value is lower for the functional networks than the random baseline networks regardless of the average degree of the nodes in the functional networks.

Using the scale-free network of **Figure 9**, we ran 100 simulations with randomly-chosen initial conditions and then formed functional networks based upon the asymptotic state of the system. As with the smaller networks, the 100 functional networks were analyzed by looking for correlations with centrality properties of the structural network. For example, the difference in the closeness centrality (in the structural network) was computed for each pair of connected nodes in the functional network, and averaged over all pairs of neighbors in the functional network. This provides a “centrality difference” measure of the mean difference in closeness centrality of connected neighbors in the functional network:

$M_{C}=\frac{\sum_{e_{ij}\in ℰ}|C_{i}-C_{j}|}{\left|ℰ\right|},$ (14)

where *ℰ*={*e*_*ij*_| nodes *i* and *j* are neighbors in the functional network}, |*ℰ*| is the total number of edges, and *C*_*i*_ is the closeness centrality in the structural network for node *i*. This provides an average measure of how far apart functionally connected neighbors are from one another in terms of the closeness centrality of the structural network. We defined similar measures for the betweenness centrality, *M*_*B*_, and eigenvector centrality, *M*_*E*_. **Figure 10A** shows *M*_*C*_ values for all 100 functional networks (blue points). To interpret these data, we reassigned all of the edges of each functional network randomly, thereby constructing an Erdős-Rényi random network as a “random baseline” for each functional network (19). We then calculated the centrality difference for this baseline network. The result is shown as orange points in **Figure 10A**. It is evident that the *M*_*C*_ values for the actual functional networks (median of 0.02) are much lower than those for the random baseline networks (median of 0.06). The implication of this is that neighboring nodes in the functional network are more likely to have similar closeness centrality than would be expected from random neighbor selection. A similar comparison was made using betweenness centrality and eigenvector centrality difference measures and the results were similar; functionally connected nodes are more likely to have similar betweenness centrality and eigenvector centrality than would occur through random neighbor selection (**Figures 10B, C**). This is true regardless of the average degree of the nodes in the functional network (and therefore the number of edges in the network), as shown for closeness centrality in **Figure 10D**.

**Figure 11:**
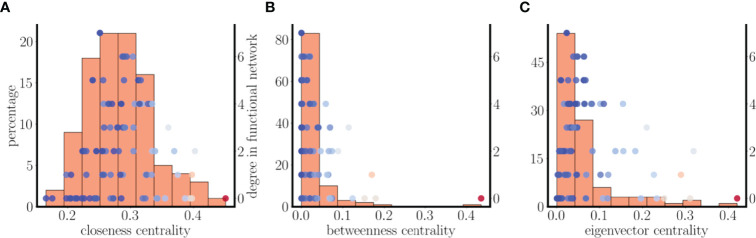
Relationship between centrality distributions and functional and structural degrees of the nodes in the random scale-free network of **Figure 9**. **(A)** Histogram of closeness centrality of nodes in the structural network (bars). The superimposed points are a scatter plot of the functional degree (for one set of initial conditions) versus the closeness centrality for each node, while the color of the points indicates the structural degree (hot colors indicate high degree). The nodes with highest functional degree are near the mean of the centrality distribution. **(B)** The distribution and scatter plot are left-skewed in terms of betweenness centrality. **(C)** There is little overlap of the eigenvector distribution and functional degree scatter plot.

Investigating the relationship between centrality and functional coupling further, we next plotted histograms of the closeness, betweenness, and eigenvector centralities of the nodes in the structural network (bars in **Figure 11**). Superimposed on these histograms are scatter plots showing the functional degrees of each of the nodes (for a single set of initial conditions) versus node centrality. There is a clear overlap between the functional degree distribution and the closeness centrality distribution, and to a lesser extent the betweenness and eigenvector centrality distributions. Another key observation is that the nodes with the highest functional degree are near the means of the centrality distributions. That is, the functional hubs are very similar to one another in terms of these three centrality measures.

**Figure 11** has one more piece of information. The points shown are color coded according to their structural degree, with hotter colors indicating higher degree. It is apparent that while the nodes with higher structural degree have high centrality values, they do not have high functional degree. In fact, they are far out on the centrality distributions, while nodes with the highest functional degree are near the means of these distributions. This demonstrates that not only are functional hubs not structural hubs, they are also not the nodes with the highest centrality.

**Figure 12:**
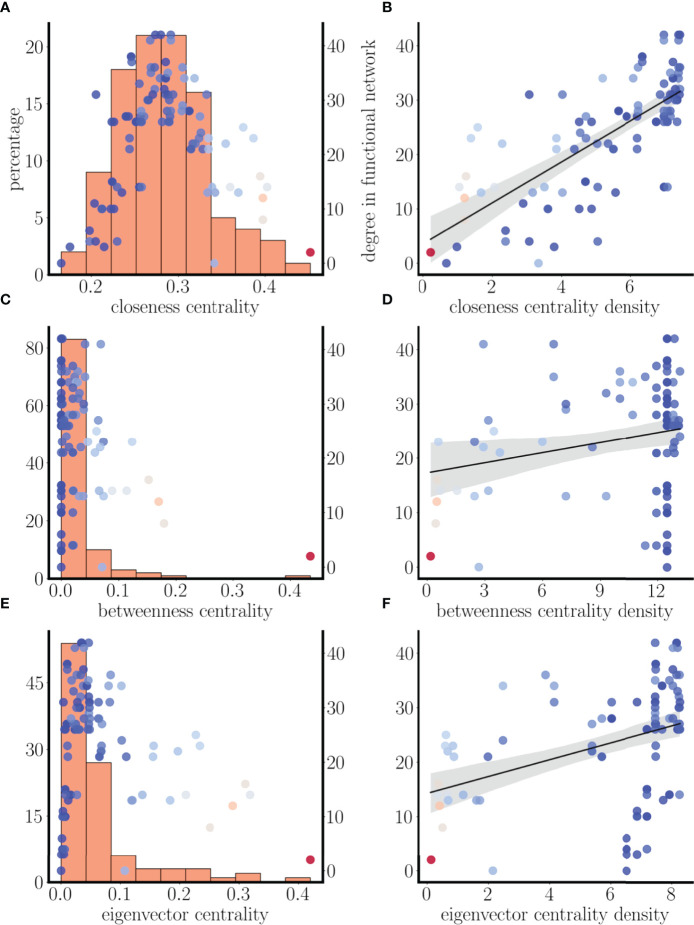
Positive correlation between functional degree and closeness centrality with similarity threshold of *S*_*ij*_>0.85. **(A, C, E)** As in **Figure 11**, but with a lower similarity threshold. **(B, D, F)** Scatter plots of degree of the nodes in the functional network vs. closeness, betweenness, and eigenvector centrality densities. For this set of initial conditions, there is a correlation of 0.72 with p-value <0.005 for closeness centrality density, but no correlation with the other measures of centrality.

In the functional networks examined thus far, two nodes were connected only if the bursting time courses almost completely overlapped, so that an edge was drawn between nodes *i* and *j* if *S*_*ij*_>0.99, where *S*_*ij*_ is the similarity metric defined in Eq. 13. This high threshold guarantees almost complete synchrony. If this threshold is relaxed somewhat, then there will be more edges in the functional network. How does this change the results from **Figure 11**? This calculation was performed on the same simulation data used in that figure, but the functional network was constructed by drawing an edge between nodes *i* and *j* if *S*_*ij*_>0.85. The functional degrees of the nodes are plotted vs. their structural network centrality in **Figures 12A, C, E**, superimposed with the structural centrality histograms from before. Once again, there is considerable overlap of the scatter plot with the closeness centrality histogram in panel A, but less of an overlap for the other two centrality measures. To make this more precise, we plotted the functional degree of each node vs. the centrality densities in **Figures 12B, D, F**. In the case of closeness centrality, there is a clear positive correlation between the functional degree and the closeness centrality density (for 57 out of 100 different sets of initial conditions tested the correlation coefficient was >0.5 with p-value <0.005). In contrast, there was no apparent correlation between the functional degree and the betweenness or eigenvector centrality (correlation coefficient never above 0.5 with any of the 100 sets of initial conditions). This suggests that the closeness centrality density is the best predictor for the functional degree of a node. Nodes with the highest closeness centrality density (i.e., those most like other nodes in terms of closeness) are most likely to be highly connected in the functional network.

## 4 Discussion

We have demonstrated how patterns of electrical coupling between model pituitary cells exhibiting pseudo-plateau bursting influence the synchronization of those cells. We first showed that, contrary to intuition, antiphasic bursting of two coupled cells is a stable state of the system (**Figure 2**). Furthermore, this antiphasic state is bistable with a synchronous state, so the cells could tend towards one state or the other, depending on the initial conditions. The antiphasic pseudo-plateau bursting is reminiscent of antiphasic spiking that was shown for model neurons (17), or antiphasic spiking within the bursts of plateau bursting cells (20), with weak electrical coupling. In essence, this demonstrates that electrical coupling between cells does not necessarily make the cells behave similarly, as might be expected (though they do if the coupling conductance is large, as shown in **Figure 3**). It is this counter-intuitive behavior that underlies all of the other results of this study, beginning with the finding that while there is a fully synchronized asymptotic state for three coupled cells, there are also additional asymptotic states in which the cells are not synchronized. In some cases the two cells that synchronize are not electrically coupled, while the cell that they are coupled to bursts out-of-phase with these two (**Figure 4**). This result was extended to small center/satellite networks, where it was found that the center cell is the least likely to be synchronized with other cells (**Figure 5**), again contrary to intuition. Moving on to multi-arm networks, we found evidence that the network centrality of nodes influenced whether or not they would synchronize (**Figures 6**, **7**). Changing the centrality of a node by adding or removing an edge to the structural network can have a large impact on the synchronization of that node with other nodes (**Figure 8**).

The small network findings demonstrate the difficulty of discerning how weak structural coupling impacts cell synchronization, i.e., the connectivity of the functional network. In the second part of the study we used a single large network to address a related question: What does the functional network tells us about the underlying structural network? Using a random scale-free structural network (**Figure 9**) and 100 functional networks determined using different sets of initial conditions, we found neighboring nodes of the functional network are more likely to have similar closeness, betweenness, and eigenvector centrality values than would be expected from random edge placement (**Figure 10**). An examination of one such functional network (obtained using a single set of initial conditions) demonstrated that nodes with high degree in the functional network tend to have high closeness centrality density (**Figure 11**), particularly if the threshold for synchronization is relaxed, which creates denser functional networks. In this case, there is a clear positive correlation between functional degree and closeness centrality density (**Figure 12**).

**Figure 13:**
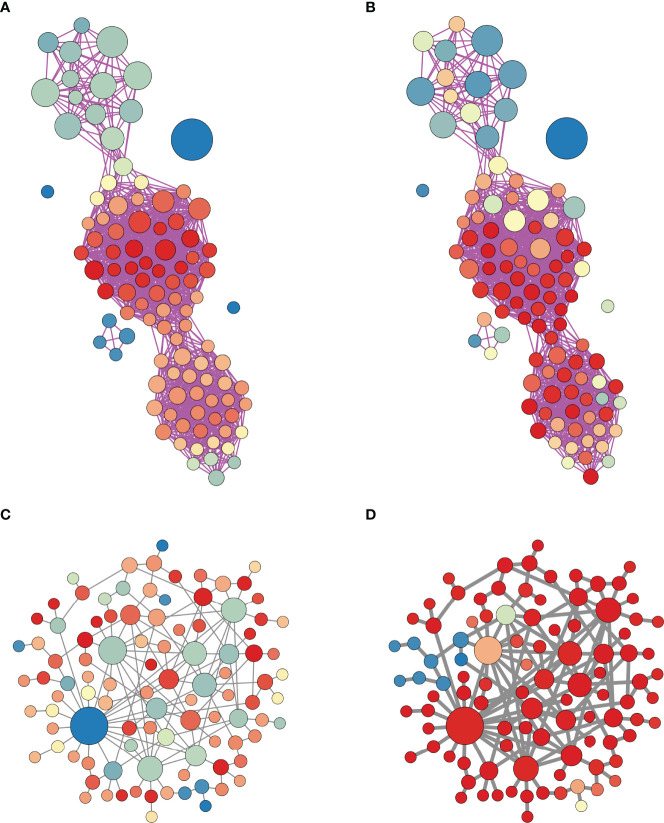
Hybrid structural-functional network representations of the scale-free network of **Figure 9**. In all panels, the node size represents its degree in the structural network. **(A)** The edges represent functional coupling (synchronization) between nodes, where *S*_*ij*_>0.85 is required for coupling between nodes *i* and *j*. The color of a node indicates functional degree, i.e., the number of nodes it synchronizes with. Hotter colors indicate higher degree. **(B)** The edges again represent functional coupling, but now the color of a node indicates its closeness centrality in the structural network. Hotter colors indicates higher centrality. **(C)** The edges now represent structural connectivity in the same network (but displayed differently to emphasize structure instead of function). The node color represents functional degree. **(D)** When the strength of coupling is increased by a factor of 5 to *g*_*c*_=10 pS (thicker edges), the network becomes much more synchronized.

These results are summarized in **Figure 13A, B**, which shows information-rich views of the 100-node scale-free network used in the latter part of the paper. The size of the nodes in the diagrams indicate their structural degree, i.e., how many other nodes they are electrically coupled to. The biggest nodes are the structural hubs of the network. The edges between the nodes indicate functional coupling, i.e., synchronization (*S*_*ij*_>0.85 for functional coupling). In panel A, the color of the nodes indicates the functional degree. The largest nodes, the structural hubs, have much less functional connectivity than the nodes with small structural degree. In fact, the node with highest structural degree is not functionally connected to any node. The functional network is most dense around clusters of nodes with low structural degree. In panel B, the node color reflects the closeness centrality density in the structural network. Many of the red nodes in this network are also red in the functional representation of panel A; the nodes with highest functional degree tend to have similar closeness centrality.

Our finding that nodes with the highest functional connectivity are not nodes with the highest structural connectivity is in contrast to a study performed on developing hippocampal networks. In this network, as in many developing networks, all synaptic connections are excitatory and the electrical activity consists of bursts of spiking throughout the neural population. GABAergic neurons are present, but because the intracellular chloride concentration is abnormally high during the early developmental stage, the neurotransmitter acts to depolarize postsynaptic cells (21). Unlike the case of electrical coupling, the synaptic coupling is unidirectional, giving rise to directed structural networks. The coupling is largely random, since this is early in development, before synaptic pruning has occurred. The study found that many of the highly connected neurons in the functional network were GABAergic, rather than glutamatergic, neurons. They also found that perturbing the electrical activity of many of these GABAergic neurons had a significant effect on network activity (the determining factor for their use of the term “functional hub”), unlike the highly functionally-connected glutamatergic neurons. Those neurons that they characterized as functional hubs were also shown to have direct synaptic connections to a large number of other neurons. In contrast, cells that were not functional hubs had much lower connectivity. Thus, the functional hubs were also found to be structural hubs (22).

We constructed our large network to have a power law degree distribution, so that there would be structural hubs. Indeed, one would assume that scale-free functional networks most likely arise from scale-free structural networks. We found that this was in fact not the case; functional hubs are not typically structural hubs. Therefore, one should not assume that scale-free structural networks are needed for scale-free functional networks, at least in the context of pituitary networks mediated by electrical coupling.

We have focused on the case of weak coupling, with coupling conductance between two cells of *g*_*c*_=2 pS. This small value was chosen since it leads to functional networks with only moderate connectivity, which is the case for pituitary lactotrophs (14) and somatotrophs (12) in pituitary slices. This is in contrast to the electrical coupling of *β*-cells within pancreatic islets, where the strong coupling (measurements and calculations range from 100 pS (23, 24) to 170 pS (25) between two cells) ensures synchronization among the *β*-cells, as has been observed both *in vitro* and *in vivo* (26, 27). The gap junctions that produce electrical coupling between cells can be rapidly produced, trafficked to the membrane and recycled in a process regulated through phosphorylation (28). The changes in electrical coupling due to changes in physiological state can be quite dramatic, as was demonstrated in lactotroph networks in pituitary slices from mice. The functional network connectivity of these cells was greatly increased in pituitaries from lactating animals and the increase in functional connectivity was still present 3 months after weaning. This increase was parallel to an increase in structural connectivity among the cells (14). A similar increase in functional connectivity was observed among mouse somatotrophs in response to application of growth hormone releasing hormone (12) and in structural connectivity during and after puberty (16). In our 100-node network model, we replicated increases in structural connectivity in two ways. First, we increased the strength of coupling between connected cells without increasing the number of edges. **Figure 13C** shows the network where the edges represent structural coupling, while size of the node represents its structural degree, and the color represents its functional degree. When the gap-junctional coupling was increased by a factor of 5, represented by thicker edges, there was a dramatic increase in network synchronization; almost all nodes are red, so almost all cells are synchronized **Figure 13D**. We also simulated increased structural connectivity by randomly adding edges. When the number of edges was increased by a factor of 5 we saw a similar dramatic increase in the level of network synchronization (not shown). Thus, by maintaining a default state of weak electrical coupling among cells, there is great potential to change the behavior of the system dramatically in response to important stimuli.

Because our focus was on the effects of electrical coupling in the generation of different functional networks, we simplified other factors as much as possible. In particular, we considered a homogeneous population of cells, each of which exhibits pseudo-plateau bursting when uncoupled. This corresponds to the activated hormone-secreting state, in contrast to a quiescent state or a tonic spiking state in which the rate of secretion is much lower (1, 3). In fact, there is a great deal of heterogeneity in the spiking patterns of endocrine pituitary cells, due both to heterogeneity in intrinsic parameters such as ion channel conductances (29, 30) and to channel noise that is substantial in these small cells (31). When cell-to-cell heterogeneity is incorporated, a large number of additional questions can be asked. For example, how influential will electrical coupling be in converting spiking cells to bursting, or vice versa? Does a structural hub have an outsized influence on its neighbors, pushing them towards similar behaviors, or is a hub more likely to conform to the spiking patterns of the majority of its many neighbors? More generally, how does network structure influence the ability of activating hormones to produce a unified positive response that results in substantial pulses of hormone release? We are currently examining these questions, which will be the focus of a future publication. Preliminary findings suggest that the task of inferring structural connectivity from functional data is even more daunting, since the native spiking pattern of each node (i.e., whether it is a spiker, burster, or some combination) affects the impact that that cell has on its neighbors. Also, that cell’s spiking pattern can change once coupled to neighbors.

Finally, we reiterate the difficulty of drawing conclusions about the structural network from the functional network. If two cells are synchronized, and thus neighbors in the functional network, that does not imply that they are electrically coupled to one another (i.e., neighbors in the structural network). In fact, there may be many intervening cells. Also, one should not conclude that two unsynchronized cells are uncoupled; they could be neighbors in the structural network. Whether two cells are synchronized or not may depend on the initial conditions, which means, from a biological viewpoint, that a perturbation to the system can cause a long-lasting change in the synchronization status. Finally, silencing or removal of a functional hub may have little effect on the dynamics of the network, because the functional hubs are not structural hubs in the case of weakly electrically coupled networks. Overall, then, it would be a mistake to attempt to draw too much structural information from an analysis of the functional network of this type.

## Data Availability Statement

Publicly available datasets were analyzed in this study. This data can be found here: www.math.fsu.edu/~bertram/software/pituitary.

## Author Contributions

MF and RB contributed to conception and design of the study. MF performed the simulations and statistical analysis. RB wrote the first draft of the manuscript. MF and RB wrote sections of the manuscript. All authors contributed to manuscript revision, read, and approved the submitted version.

## Funding

This work was partially supported by NSF grant DMS 1853342 to RB.

## Conflict of Interest

The authors declare that the research was conducted in the absence of any commercial or financial relationships that could be construed as a potential conflict of interest.

## Publisher’s Note

All claims expressed in this article are solely those of the authors and do not necessarily represent those of their affiliated organizations, or those of the publisher, the editors and the reviewers. Any product that may be evaluated in this article, or claim that may be made by its manufacturer, is not guaranteed or endorsed by the publisher.
